# Resistance is not Futile: It Shapes Insecticide Discovery

**DOI:** 10.3390/insects5010227

**Published:** 2014-01-23

**Authors:** Margaret C. Hardy

**Affiliations:** 1 Institute for Molecular Bioscience, The University of Queensland, Queensland 4072, Australia; E-Mail: m.hardy@imb.uq.edu.au; Tel.: +61-7-3346-2018; Fax: +61-7-3346-2101; 2 Queensland Alliance for Agriculture and Food Innovation, The University of Queensland, Queensland 4072, Australia

**Keywords:** agriculture, arbovirus vectors, biosecurity, conservation, malaria

## Abstract

Conventional chemical control compounds used for the management of insect pests have been much maligned, but still serve a critical role in protecting people and agricultural products from insect pests, as well as conserving biodiversity by eradicating invasive species. Although biological control can be an effective option for area-wide management of established pests, chemical control methods are important for use in integrated pest management (IPM) programs, as well as in export treatments, eradicating recently arrived invasive species, and minimizing population explosions of vectors of human disease. Cogitated research and development programs have continued the innovation of insecticides, with a particular focus on combating insecticide resistance. Recent developments in the fields of human health, protecting the global food supply, and biosecurity will be highlighted.

## 1. Introduction

Each year, insects vector 219 million cases of malaria [1], damage 10–30% of the world’s food supply [2], and invasive species cause $20 billion in damage [3]. Despite the impact of insects on food production, human health, and the environment, we still lack sustainable management technologies.

Indiscriminate spraying of chemical insecticides as a standalone management plan for insect pests is increasingly rare. Formulation chemistries and application technologies are more sophisticated, and allow for the increased use of baits and attractants [4,5]. The widespread adoption of other approaches are still in regulatory or scientific development, including genetically engineered plants [6,7,8] or insects [9], nanotechnology-based pesticide application methods [10,11,12], malaria vaccines [13], and RNAi‑mediated protection from insect pests [14]. Amid concerns about the safety of some biocontrol programs where nonnative natural enemies are introduced [15], and the increase in a desire for chemical control options that are compatible with integrated pest management (IPM) programs, there is a growing need for novel, selective insecticides [16]. However, the most pressing reason for the need to discover and appropriately characterize selective compounds is the global development of insecticide resistance. 

The mechanisms of insecticide resistance are generally well described. The two most common forms of resistance are target-site modifications, which cause a mutation in the target that results in the insecticide no longer binding to its target; or, enzyme-based resistance, which is caused by enhanced or modified activities of detoxification enzymes (including esterases, oxidases, or glutathione S-transferases) that prevents the insecticide from reaching its molecular target. Several comprehensive general reviews on molecular mechanisms of insecticide resistance have been published [17,18,19,20,21,22,23]. A variety of reviews have already been published detailing specific resistance mechanisms for mosquitoes [24,25,26,27,28,29] and agricultural pests [30,31,32,33]. Recently the first instance of insecticide resistance mediated by an intestinal symbiont was reported [34]. In this case, the soil-dwelling symbiotic bacteria in the genus *Burkholderia* degrade the organophosphate insecticide fenitrothion to an innocuous carbon source via two less toxic intermediates (3-methyl-4-nitrophenol and methylhydroquinone). Additional, ‘weaker’ adaptations including penetration and behavioral modifications may be present but difficult to detect.

Given the key and mutable role of insecticides in vector control, IPM programs and crop pest management, and for biosecurity and conservation efforts a clear review of insecticide resistance is opportune. In order to provide an inclusive snapshot of the international insecticide climate, national databases were mined for information and the results synthesized. 

## 2. Experimental Section

The goal of this work is to provide a comprehensive review of the global impact of insecticide resistance, with a specific focus on insects that are relevant to the fields of medicine, biosecurity and conservation, and agriculture. PubMed, the Insecticide Resistance Action Committee (IRAC) e‑classification method, the Arthropod Pesticide Resistance Database, IR Mapper, the Food and Agriculture Organization, and Invasive.org were mined for data on insecticide classification and insecticide resistance. Brief descriptions of the methods used to create each table or figure follow.

An effective way of categorizing the active ingredient (AI) of insecticides is into groups based on the mechanism of action of a compound. IRAC was formed to provide “a coordinated crop protection industry response to prevent or delay the development of resistance in insect and mite pests,” [35] The IRAC scheme groups insecticides with a similar mode of action together [36,37]. This method has provided for at least 27 different insecticide categories based on mode of action (Table 1).

However, grouping AIs based on mode of action alone neglects to provide a complete overview of the narrowness of the current insecticide discovery landscape. The mechanism of action is a critical feature of any pesticide, and as a result the mechanism, as well as the target, should be clearly explored for novel insecticidal compounds. With entirely novel chemistries it is possible the mechanism cannot be easily determined. If the AIs were grouped only by their molecular target, all the compounds can be re‑classified into ten categories (Table 2). However, metabolic mechanisms may act across mode of action groups (e.g., esterases against pyrethroids and organophosphates).

**Figure 1:**
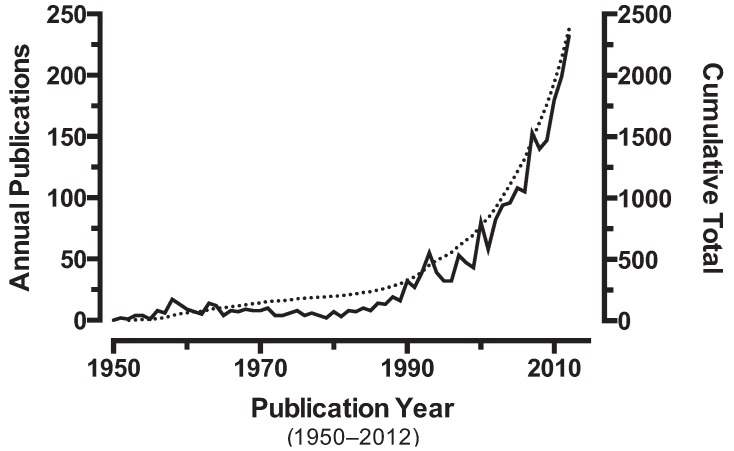
The annual number of scientific papers published with “insecticide AND resistance” in the title/abstract abstracted in PubMed. The overlaid curve (dashed line) shows the cumulative total number of publications on insecticide resistance.

Figure 1 was created using data collected from PubMed [38], using the terms “insecticide AND resistance” in the title/abstract from 1950–2012. PubMed was selected because the database broadly encompasses research relevant to the fields of medical and veterinary entomology, as well as agriculture and biosecurity.

**Figure 2:**
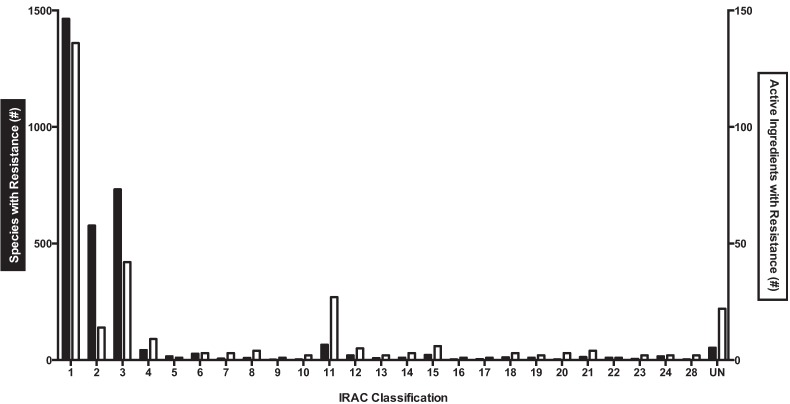
The number of species (black bar, right y-axis) or active ingredients (white bar, left y-axis) with documented insecticide resistance for each IRAC class (based on the molecular target). Only class 25 (mitochondrial complex II electron transport inhibitors) had no documented resistance; classes 26 and 27 remain unallocated. Note the different scale for the left and right y-axis.

**Figure 3:**
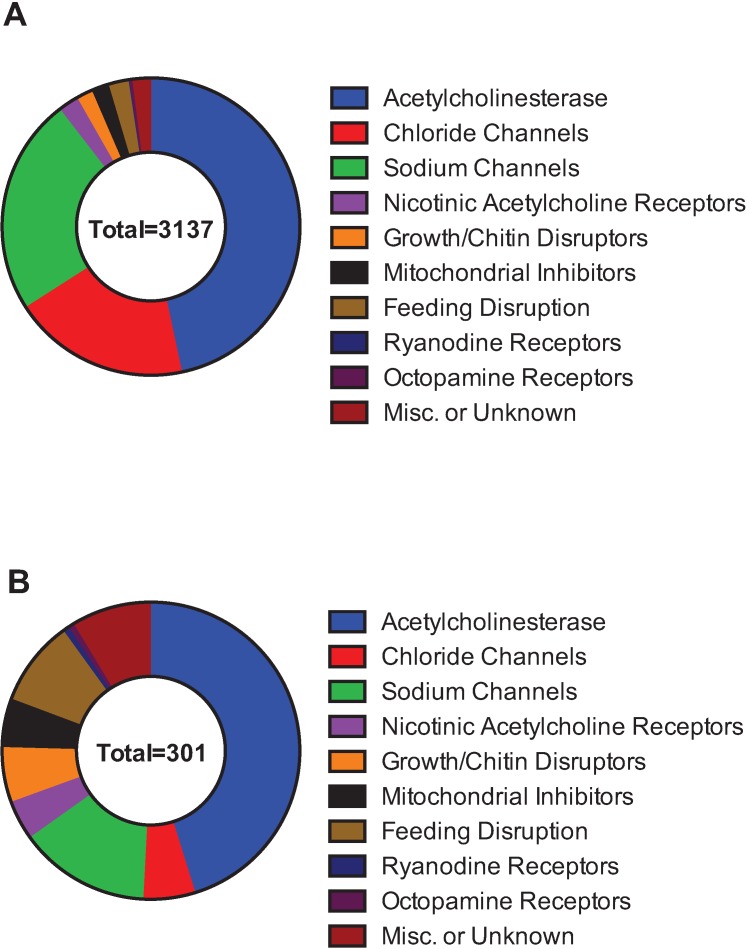
Insecticide resistance, by the cases of resistant insect species (**A**) or the number of active ingredients with documented incidents of insecticide resistance (**B**). Data compiled from the Arthropod Pesticide Resistance Database and IRAC.

For Figure 2 and Figure 3, insecticide classifications are based on IRAC groupings [37], and resistance information comes from the Arthropod Pesticide Resistance Database [39].

The World Health Organization (WHO) Pesticide Evaluation Scheme [40] is comprised of only four classes of insecticides: pyrethroids, organochlorines, carbamates, and organophosphates. In order to determine the impact of insecticide resistance on vectors of malaria, IR Mapper [41] was used to visualize global patterns. 

Data for Figure 6 and Table S2 were collected from Invasive.org [42], a joint project of the Center for Invasive Species and Ecosystem Health and the United States Department of Agriculture Animal and Plant Health Inspection Service Plant Health, Plant Protection and Quarantine program (UDSA APHIS PPQ).

The Food and Agriculture Organization (FAO) of the United Nations [43] provided data and maps for the use of pesticides on arable land and permanent crops (tonnes of active ingredient per 1000 Ha) by country (Figure 5) and the top importing countries of hazardous pesticides (Figure 7). 

## 3. Results and Discussion

### 3.1. Insecticides in Context

One critical metric for research is the publication record; publications reflect funding, interest, and scientific merit [44]. However, it is unclear whether this represents the extent and growth over time of resistance or our recognition of the problem. The interest generated by insecticide resistance over time can be tracked by following the rate of publication for manuscripts on the topic (Figure 1).

Several major national and international organizations have existing toxicity classification schemes for pesticides, but rely on ranking pesticides in terms of their toxicity in vertebrates with the aim of protecting human health (Table 1). 

**Table 1:** An overview of the insecticide classification schemes in use today. Note all but the two schemes in the shaded rows base their classification on vertebrate toxicity.

Organization	Standard	Year Enacted
European Union	Council Directive 67/548/EEC	1967
US Environmental Protection Agency	Federal Insecticide, Fungicide, and Rodenticide Act (FIRFA)	1972
World Health Organization	Recommended Classification of Pesticides by Hazard	1975
IOBC ^1^	Pesticides Side-Effects Standards	1985
IRAC ^2^	Mode of Action Classification Scheme	2001
United Nations	Globally Harmonized System of Classification and Labeling of Chemicals	2002

^1^ IOBC: International Organization for Biological and Integrated Control; ^2^ IRAC: Insecticide Resistance Action Committee.

A more parsimonious classification is based on the chemical class and molecular target, as these can be experimentally validated in the target organism (rather than extrapolated from model systems, as human toxicity is usually inferred from rats or mice in the other schemes). Further, classification based on molecular target allows structurally dissimilar compounds to be appropriately monitored in resistance management programs, and for vertebrate toxicity to be assessed from an informed position.

IRAC, and its equivalent groups FRAC (for fungicides) and HRAC (for herbicides), are a global industry consortium aimed at providing information to prevent and delay the onset of resistance [36]. The IRAC scheme groups insecticides together based on the mode of action, whereas the IOBC assesses the toxicity of compounds in beneficial non-target insects (pollinators and natural enemies). Using the IRAC classification scheme, 27 different classes of insecticide have been delineated; cross-resistance can develop against any AIs within the same mechanism of action (Table S1). 

Within a single molecular target different compounds can have a variety of mechanisms. This is particularly so for ion channels, which can be acted upon in a variety of ways—including by agonists, antagonists, modulators, and inhibitors. If the classification is based on the molecular target generally, only ten categories remain (Table 2). 

**Table 2:** Classification of insecticides based on molecular target.

By Molecular Target	By IRAC Category
Acetylcholinesterase	1
Chloride Channels	2, 6
Sodium Channels	3, 22
Nicotinic Acetylcholine Receptors	4, 5, 14
Growth/Chitin Disruptors	7, 10, 15, 16, 17, 18, 23
Mitochondrial Complex Electron Transport Inhibitors	12, 13, 20, 21, 24, 25
Feeding Disruption	9, 11
Ryanodine Receptors	28
Octopamine Receptors	19
Miscellaneous or Unknown	8, UN

More selective insecticidal compounds are increasingly desirable for commercial and environmental reasons, and so the description of novel insect channels and receptors could become the rate-limiting step in mechanism determination. Basic research into insect physiology is essential in order to continue to prove the safety and efficacy of new compounds, and provide for more options to combat insecticide resistance. Chitin synthesis inhibitors [45], GABA and glutamate receptors [46] are examples of highly selective technologies which inherently target arthropods exclusively.

### 3.2. Insecticide Resistance and Cross Resistance

The implications of cross-resistance between AIs with similar modes of action are far-reaching, and are a direct result of the lack of novel molecular targets for insecticides. Neurotoxic insecticidal compounds have long been sought because of their ideal properties of efficacy and safety for pest insect management [46]. However, one of the major factors to consider with the development and application of insecticides is the vertebrate toxicity of the compounds being applied. Finally, the levels of pesticide residues must be maintained below a minimum threshold which is a particular challenge in predominantly agrarian societies [47], particularly because the threshold is often determined by analytical sensitivity, rather than logically. 

In order to determine the scale of documented insecticide resistance across species, data from IRAC and the Arthropod Pesticide Resistance Database was combined (Figure 2). In total, 3,137 species had proven cases of insecticide resistance against 301 different active ingredients. 

Using the information from Table 2, another way to classify the compounds is based on molecular target. Insecticides that target acetylcholinesterase (class 1), GABA-gated chloride (classes 2, 6) and sodium channels (classes 3, 22) make up 90% of the cases of resistant species and 65% of the active ingredients with resistance (Figure 3A,B). Insecticides that target acetylcholinesterase (those in IRAC class 1A and 1B) respectively comprise 47% and 45% of the resistant species and AIs. 

Although the same insect species can demonstrate resistance to several insecticide classes or active ingredients, three insecticides make up 90% of the most commonly documented cases of resistance. 

In order to further examine the impact of insecticide resistance, three case studies are provided on human health, the global food supply, and for biosecurity and conservation efforts.

#### 3.2.1. Human Health

Mosquitoes are effective vectors of human disease-causing agents, including parasites (like malaria-causing *Plasmodium* and the filariasis worm that causes elephantiasis), and arboviruses (including viruses that cause dengue fever, Eastern Equine Encephalitis, and Ross River Fever). Despite the efforts of integrated vector management programs there is still a lack of effective compounds to manage mosquito populations below critical infection thresholds [48]. 

The World Health Organization Pesticide Evaluation Scheme (WHOPES) is designed to provide defined, safe, effective treatment options for vectors of human disease, most importantly for mosquitoes that vector malaria. Because human health is at risk, efficacy and safety are paramount. WHOPES only includes a handful of active ingredients from four classes: pyrethroids, organochlorines, carbamates, and organophosphates. These approved insecticides have remained largely unchanged since the late 1980s when etofenprox was added [24]. 

**Figure 4:**
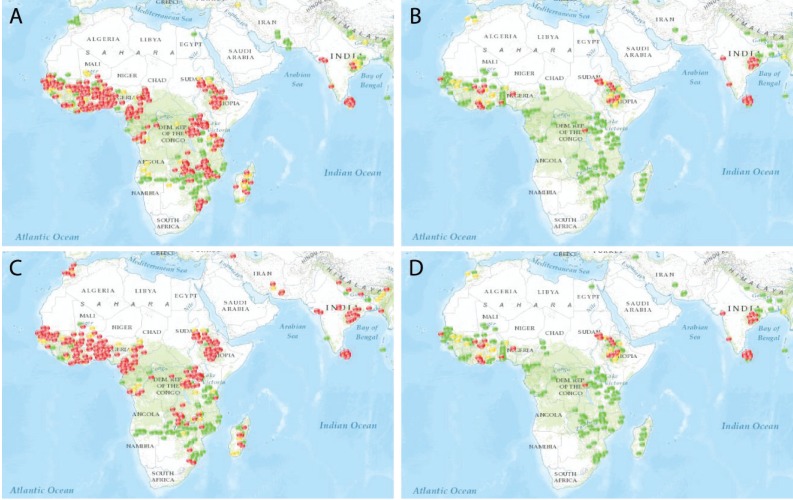
A comparison of insecticide resistance in *Anopheles* mosquitoes in areas where *Plasmodium falciparum* or *P. vivax*, or both, are endemic (from IR Mapper). Reported cases (2000–2012, based on WHO criteria) are indicated by dots: red for confirmed resistance (<90% mortality), yellow for possible resistance (90–97% mortality), or green for susceptibility (98–100% mortality). Each panel represents a different insecticide class, namely, pyrethroids (**A**), carbamates (**B**), organochlorines (**C**), or organophosphates (**D**).

To further complicate management, compounds from these insecticide classes have only two modes of action. Pyrethroids (class 3A) and organochlorines (3B) modulate the insect sodium channel; carbamates (1A) and organophosphates (1B) are both inhibitors of acetylcholinesterase. Thus, pyrethroids (Figure 4A) and organochlorines (Figure 4C) exhibit similar geographic patterns of resistance and susceptibility, as do carbamates (Figure 4B) and organophosphates (Figure 4D). 

The impact of the limited number of active ingredients is visible in the documented cases of insecticide resistance in areas where malaria is endemic. Recently, urban populations of *An. gambiae* in Nigeria have been shown to be resistant to carbamates, DDT, and deltamethrin. One mechanism of target site modification (*kdr*) confers cross-resistance to two different insecticide classes (DDT and pyrethroids) with the same mode of action (modulating sodium channels) [49]. Rotations of long‑lasting formulations of insecticides suitable for indoor residual spraying have reduced the selection pressure for resistance to non-pyrethroid insecticides, and provide a template for resistance management for malaria mosquito control programs [50].

Previous work has shown that infection with entomopathogens increases the susceptibility of resistant mosquitoes to pyrethroids, carbamates, or organochlorine insecticides [51]. Entomopathogens have been shown to be compatible with some selective registered insecticides [52], and some have exhibited synergism with conventional insecticides [53]. Entomopathogens can also be modified to produce other peptides that increase their toxicity and decrease the time-to-death [54,55,56,57]. Fusion proteins consisting of plant lectin and an insecticidal toxin can be an effective way to deliver insecticidal peptides directly into the insect hemolymph. Spider and scorpion venom peptides are particularly useful in this manner, since the fusion protein brings the neurotoxic venom peptide directly into contact with its’ molecular target in the insect nervous system [58,59,60]. 

#### 3.2.2. Global Food Supply

The main factors that affect food security are the demand for food, future trends in the food supply, and exogenous factors [61]. In addition to crops in the ground, additional losses occur due to stored product pests. Fumigation with phosphine gas is still the most commonly accepted treatment before dry storage or export [62,63]. As IPM programs are more widely adopted, selective and appropriate chemical options are needed once pests reach the treatment threshold [64]. On a global scale pesticide use has been largely decreasing, but in many countries chemical control makes up a large portion of their response to insects that endanger human health, crops and stored products, and native ecosystems.

**Figure 5:**
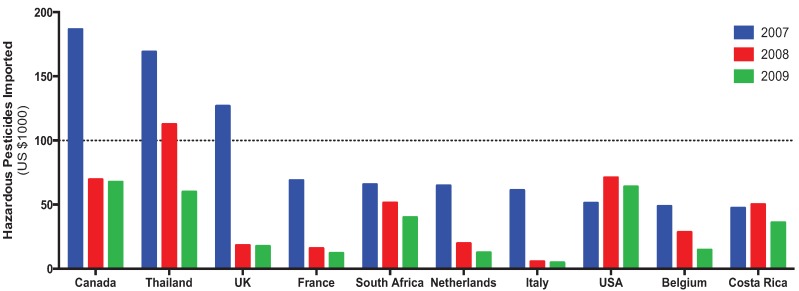
Expenditures for the top ten pesticide-importing countries (2007–2009).

The expense needed to apply pesticides is not insignificant, nor is it an exclusive problem of rich or poor countries. At least one year from 2007–2009, all of the top ten countries to import hazardous pesticides (as defined by the FAO) spent in excess of US$50,000 (Figure 5). The top three countries in 2007 (Canada, Thailand, and the United Kingdom) all decreased their usage by 2009, and only the United States increased its pesticide importation expenditure from the initial measure. On the whole, a decrease in pesticide use was seen across nine of the countries, possibly due to the adoption of IPM programs, increase use of genetically engineered crops, or pesticide regulation and deregistration.

Overall, global pesticide use decreased from 1990–2010, and that trend was also manifested in a decrease in expenditures from the countries that previously spent the most importing pesticides.

The FAO has monitored global pesticide use since 1992. If a country did not report using a particular pesticide, and there is no data from another source for that country, there will not be any value. FAOSTAT uses the following equation to calculate total pesticide consumption (Equation 1).

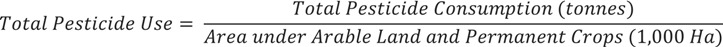
(1)


**Figure 6:**
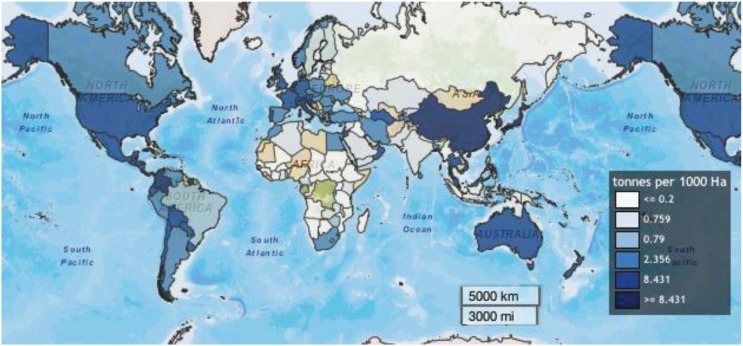
Average pesticide use from 1992–2010 on arable land and permanent crops Food and Agriculture Organization of the United Nations (FAOSTAT).

Although no data on insecticide use alone is available, hotspots of pesticide application activity are clearly visible; darker shades of blue correspond to higher reported rates of pesticide use (Figure 6).

Overall, global pesticide use decreased from 1990–2010, and that trend was also manifested in a decrease in expenditures from the countries that previously spent the most importing pesticides.

#### 3.2.3. Biosecurity and Conservation

Insecticides can be used for eradication of newly arrived invasive species and targeted approaches for the elimination of long-standing colonies. One concern is the use of broad-spectrum insecticides in island or otherwise sensitive ecosystems, particularly with social insects due to their recalcitrant pest status [65]. Based on the concept of IPM, integrated pest eradication (IPE) programs aim to systematically use several eradication tools in concert, and narrow-spectrum, ‘green’ chemical insecticides are ideal for use in IPE programs. Although the cost of eradication programs is difficult to estimate, as the pest insect is not allowed to establish and reach 100% of its potential damage levels, estimates for the eradication of invasive forest insects in New Zealand range from 2:1 to 8:1 for benefit:cost [66]. 

**Figure 7:**
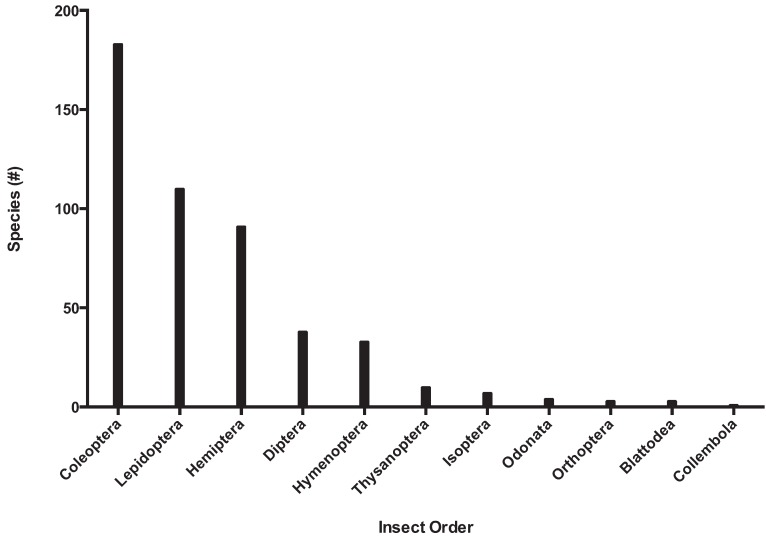
The number of invasive insect species in North America by phylogenetic order.

Biosecurity programs provide two important services: import regulations and export certification. There is significant overlap between the fields of biosecurity and conservation when considering the importance of the management and eradication of invasive insect species. In North America alone, invasive species contribute to over 40% of the listings on the United States Fish and Wildlife Service Threatened or Endangered species list [67]. Coleoptera and Lepidoptera are the most common invaders, with over 150 and 100 invasive species recorded, respectively (Figure 7). 

Hemiptera, Diptera, and Hymenoptera are also well represented, and the species from these orders are among the most economically important insect pests; a detailed table of the list by family and the number of species is available as supplementary data (Table S2).

The most successful invasive families with more than 20 species listed are Curculionidae (99 species), Cerambycidae (35), Tortricidae (26), Tephritidae and Diaspididae (22 each). All five families disproportionately affect primary producers: with the exception of curculionids, which are primarily a forestry and timber pest, the other families are notable agricultural pests. These families may be successful because of their relative size, or some other aspect of their biology or ecology that increases their probability of introduction and establishment. With the proliferation of global agricultural pests it will be increasingly important to provide management options for invasive insect pests.

From a conservation perspective, the arrival of invasive species to an ecosystem also has a direct impact on the evolution of native species, as well as on the invaders [68]. Thus, by not eradicating invasive species before they have a chance to establish we potentially make them better suited to invade another environment. Recent advances in the management of invasive species in sensitive ecosystems provide an opportunity for highly targeted, specific campaigns centered on chemical control to eradicate species before the population becomes established [65].

## 4. Conclusions

Understanding the global atmosphere of resistance is a critical step in insecticide discovery. From a molecular perspective, the mechanisms of resistance have been established for the major classes of insecticides, but more advances in basic insect physiology are needed in order to speed up the directed discovery of novel compounds. Because of their potency, safety, and efficacy, bioderived rational insecticides can serve as a valuable source of novel compounds, as well as a pharmacological probe for finding novel molecular insecticide targets. 

Using the publically available resources to classify and monitor insecticide resistance, a global picture develops. By classifying insecticides by their mechanism of action, rather than vertebrate toxicity, the development of insecticide resistance can be more accurately predicted and monitored. Using a mechanism-based classification scheme also helps to identify potential incidences of cross-resistance, which is critical for IPM programs and conventional pest management alike. The need for selective, sustainable new insecticidal compounds becomes evident when the level of resistance in vectors of human disease, crop and livestock pests, and invasive species is examined.

Current insecticide chemistries rely on a handful of molecular targets, and the danger of cross-resistance increases as the same active ingredients are used repeatedly. Despite difficulties in determining the mechanism of action for insecticides, even if the target is known, it will be critical to have a diversity of compounds registered for use. Entomology in particular is a global science, and insect pests are becoming more cosmopolitan. Looking to nature’s combinatorial chemists (arthropod venoms, botanical derivatives) provides a rational design strategy for novel compounds. With advances in electrophysiology, structural biology, and genomics, mining biological experts for new compounds will be a key component of research programs. In addition to insecticide discovery programs, additional insect physiology work to discover new molecular targets will be an important step forward. Matching these research projects with an understanding of mitigating insecticide resistance will be critical for success. 

In the future, technologies like nanoapplication of insecticides, genetically engineered crops, insects, and entomopathogens, and RNAi-mediated crop protection could become a reality. Insecticide resistance monitoring can and should implement mathematical modeling from other interdisciplinary fields, such as the development of antibiotic resistance [69]. At the moment, we need selective, sustainable chemical control options that are suitable for use in IPM programs to protect crops and livestock, as well as providing options for the eradication of invasive insects and vectors of human disease. 

## Supplementary Files

Supplementary File 1

Supplementary File 2

## References

[1] WHO Global Malaria Programme (2012). World Malaria Report.

[2] OERKE E.-C. (2005). Crop losses to pests. J. Agric. Sci..

[3] Pimentel D., Lach L., Zuniga R., Morrison D. (2000). Environmental and economic costs of nonindigenous species in the United States. Bioscience.

[4] Hardy M.C. (2011). Using selective insecticides in sustainable IPM. CAB Rev. Perspect. Agric. Vet. Sci. Nutr. Nat. Resour..

[5] Lu Y., Wu K., Jiang Y., Guo Y., Desneux N. (2012). Widespread adoption of Bt cotton and insecticide decrease promotes biocontrol services. Nature.

[6] Kennedy G.G., Romeis J., Shelton A.M., Kennedy G.G. (2008). Integration of Insect-Resistant Genetically Modified Crops within IPM Programs. Integration of Insect-Resistant Genetically Modified Crops within IPM Programs.

[7] Romeis J., Bartsch D., Bigler F., Candolfi M.P., Gielkens M.M.C., Hartley S.E., Hellmich R.L., Huesing J.E., Jepson P.C., Layton R., Quemada H., Raybould A., Rose R.I., Schiemann J., Sears M.K., Shelton A.M., Sweet J., Vaituzis Z., Wolt J.D. (2008). Assessment of risk of insect-resistant transgenic crops to nontarget arthropods. Nat. Biotechnol..

[8] Bradford K.J., Van Deynze A., Gutterson N., Parrott W., Strauss S.H. (2005). Regulating transgenic crops sensibly: Lessons from plant breeding, biotechnology and genomics. Nat. Biotechnol..

[9] Wise de Valdez M.R., Nimmo D., Betz J., Gong H.-F., James A.A., Alphey L., Black W.C. (2011). Genetic elimination of dengue vector mosquitoes. Proc. Natl. Acad. Sci. U.S.A..

[10] Chaudhry Q., Scotter M., Blackburn J., Ross B., Boxall A., Castle L., Aitken R., Watkins R. (2008). Applications and implications of nanotechnologies for the food sector. Food Addit. Contam. Part A. Chem. Anal. Control. Expo. Risk Assess..

[11] Bouwmeester H., Dekkers S., Noordam M.Y., Hagens W.I., Bulder A.S., de Heer C., ten Voorde S.E.C.G., Wijnhoven S.W.P., Marvin H.J.P., Sips A.J.A.M. (2009). Review of health safety aspects of nanotechnologies in food production. Regul. Toxicol. Pharmacol..

[12] Rai M., Ingle A. (2012). Role of nanotechnology in agriculture with special reference to management of insect pests. Appl. Microbiol. Biotechnol..

[13] Seder A., Sarwar N., Gordon I.J., Holman L.A., James E.R., Billingsley P.F., Gunasekera A., Manoj A., Li M., Ruben A.J., Li T., Abraham G., Stafford R.E., Plummer S.H., Cynthia S., Novik L., Costner P.J.M., Mendoza F.H., Saunders J.G., Nason C., Richardson J.H., Davidson S.A., Richie T.L., Sutamihardja A., Fahle G.A., Kirsten E., Laurens M.B., Tewari K., Epstein J.E., Sim B.K.L., Ledgerwood J.E. (2013). Protection against malaria by intravenous immunization with a nonreplicating sporozoite vaccine. Science.

[14] Zhang H., Li H.-C., Miao X.-X. (2013). Feasibility, limitation and possible solutions of RNAi-based technology for insect pest control. Insect Sci..

[15] Louda S.M., Russell F.L. (2003). Invasiveness of some biological control insects and adequacy of their ecological risk assessment and regulation. Conserv. Biol..

[16] Drogui P., Lafrance P., Lichtfouse E. (2012). Pesticides and Sustainable Agriculture. Farming for Food and Water Security.

[17] McKenzie J.A. (2000). The character or the variation: the genetic analysis of the insecticide-resistance phenotype. Bull. Entomol. Res..

[18] ffrench-Constant R.H. (2007). Which came first: insecticides or resistance?. Trends Genet..

[19] Toda S., Komazaki S., Tomita T., Kono Y. (2004). Two amino acid substitutions in acetylcholinesterase associated with pirimicarb and organophosphorous insecticide resistance in the cotton aphid, Aphis gossypii Glover (Homoptera: Aphididae). Insect Mol. Biol..

[20] ffrench-Constant R.H., Pittendrigh B., Vaughan A., Anthony N. (1998). Why are there so few resistance-associated mutations in insecticide target genes?. Philos. Trans. R. Soc. London B Biol. Sci..

[21] Clark J.K., Scott J.G., Campos F., Bloomquist J.R. (1995). Resistance to avermectins: extent, mechanisms, and management implications. Annu. Rev. Entomol..

[22] Bloomquist J.R. (1996). Ion channels as targets for insecticides. Annu. Rev. Entomol..

[23] Soderlund D.M., Bloomquist J.R. (1989). Neurotoxic actions of pyrethroid insecticides. Annu. Rev. Entomol..

[24] Nauen R. (2007). Insecticide resistance in disease vectors of public health importance. Pest Manag. Sci..

[25] Enayati A., Hemingway J. (2010). Malaria management: past, present, and future. Annu. Rev. Entomol..

[26] Kelly-Hope L., Ranson H., Hemingway J. (2008). Lessons from the past: managing insecticide resistance in malaria control and eradication programmes. Lancet Infect. Dis..

[27] Hemingway J., Ranson H. (2000). Insecticide resistance in insect vectors of human disease. Annu. Rev. Entomol..

[28] Brogdon W.G., McAllister J.C. (1998). Insecticide resistance and vector control. Emerg. Infect. Dis..

[29] Hoy M.A. (1998). Myths, models and mitigation of resistance to pesticides. Philos. Trans. R. Soc. London B Biol. Sci..

[30] Carrière Y., Ellers-Kirk C., Hartfield K., Larocque G., Degain B., Dutilleul P., Dennehy T.J., Marsh S.E., Crowder D.W., Li X., Ellsworth P.C., Naranjo S.E., Palumbo J.C., Fournier A., Antilla L., Tabashnik B.E. (2012). Large-scale, spatially-explicit test of the refuge strategy for delaying insecticide resistance. Proc. Natl. Acad. Sci. USA.

[31] Bloomquist J.R. (1994). Cyclodiene resistance at the insect GABA receptor/chloride channel complex confers broad cross resistance to convulsants and experimental phenylpyrazole insecticides. Arch. Insect Biochem. Physiol..

[32] Martin T., Chandre F., Ochou O.G., Vaissayre M., Fournier D. (2002). Pyrethroid resistance mechanisms in the cotton bollworm Helicoverpa armigera (Lepidoptera: Noctuidae) from West Africa. Pestic. Biochem. Physiol..

[33] George J.E., Pound J.M., Davey R.B. (2004). Chemical control of ticks on cattle and the resistance of these parasites to acaricides. Parasitology.

[34] Kikuchi Y., Hayatsu M., Hosokawa T., Nagayama A., Tago K., Fukatsu T. (2012). Symbiont-mediated insecticide resistance. Proc. Natl. Acad. Sci. USA.

[35] IRAC. http://www.irac-online.org/about/irac/.

[36] Nauen R., Elbert A., Mccaffery A., Slater R., Krämer W., Schirmer U., Jeschke P., Witschel M. (2012). Modern Crop Protection Compounds, Volumes 1-3.

[37] IRAC. http://www.irac-online.org/eClassification/#.

[38] PubMed. http://www.ncbi.nlm.nih.gov/pubmed.

[39] Arthropod Pesticide Resistance Database. http://www.pesticideresistance.com/.

[40] WHOPES. http://www.who.int/whopes/en/.

[41] IR Mapper. http://www.irmapper.com/.

[42] Invasive.org. http://www.invasive.org.

[43] FAOSTAT. http://faostat3.fao.org/.

[44] Renear A., Palmer C. (2009). Strategic reading, ontologies, and the future of scientific publishing. Science.

[45] Merzendorfer H. (2013). Chitin synthesis inhibitors: old molecules and new developments. Insect Sci..

[46] Casida J.E., Durkin K.A. (2013). Neuroactive insecticides: targets, selectivity, resistance, and secondary effects. Annu. Rev. Entomol..

[47] Abhilash P.C., Singh N. (2009). Pesticide use and application: an Indian scenario. J. Hazard. Mater..

[48] Enayati A., Hemingway J. (2010). Malaria management: past, present, and future. Annu. Rev. Entomol..

[49] Oduola A.O., Idowu E.T., Oyebola M.K., Adeogun A.O., Olojede J.B., Otubanjo O.A., Awolola T.S. (2012). Evidence of carbamate resistance in urban populations of Anopheles gambiae s.s. mosquitoes resistant to DDT and deltamethrin insecticides in Lagos, South-Western Nigeria. Parasit. Vectors.

[50] Hemingway J., Vontas J., Poupardin R., Raman J., Lines J., Schwabe C., Matias A., Kleinschmidt I. (2013). Country-level operational implementation of the Global Plan for Insecticide Resistance Management. Proc. Natl. Acad. Sci. USA.

[51] Farenhorst M., Mouatcho J.C., Kikankie C.K., Brooke B.D., Hunt R.H., Thomas M.B., Koekemoer L.L., Knols B.G.J., Coetzee M. (2009). Fungal infection counters insecticide resistance in African malaria mosquitoes. Proc. Natl. Acad. Sci..

[52] Negrisoli A.S., Garcia M.S., Barbosa Negrisoli C.R.C. (2010). Compatibility of entomopathogenic nematodes (Nematoda: Rhabditida) with registered insecticides for Spodoptera frugiperda (Smith, 1797) (Lepidoptera: Noctuidae) under laboratory conditions. Crop Prot..

[53] Wraight S.P., Ramos M.E. (2005). Synergistic interaction between Beauveria bassiana- and Bacillus thuringiensis tenebrionis-based biopesticides applied against field populations of Colorado potato beetle larvae. J. Invertebr. Pathol..

[54] Lu D., Pava-Ripoll M., Li Z., Wang C. (2008). Insecticidal evaluation of Beauveria bassiana engineered to express a scorpion neurotoxin and a cuticle degrading protease. Appl. Microbiol. Biotechnol..

[55] St. Leger R., Wang C. (2010). Genetic engineering of fungal biocontrol agents to achieve greater efficacy against insect pests. Appl. Microbiol. Biotechnol..

[56] Wang C., St Leger R.J. (2007). A scorpion neurotoxin increases the potency of a fungal insecticide. Nat. Biotechnol..

[57] Fang W., Vega-Rodriguez J., Ghosh A.K., Jacobs-Lorena M., Kang A., St Leger R.J. (2011). Development of transgenic fungi that kill human malaria parasites in mosquitoes. Science.

[58] Fitches E.C., Pyati P., King G.F., Gatehouse J.A. (2012). Fusion to snowdrop lectin magnifies the oral activity of insecticidal ω-Hexatoxin-Hv1a peptide by enabling its delivery to the central nervous system. PLoS One.

[59] Fitches E.C., Bell H.A., Powell M.E., Back E., Sargiotti C., Weaver R.J., Gatehouse J.A. (2010). Insecticidal activity of scorpion toxin (ButaIT) and snowdrop lectin (GNA) containing fusion proteins towards pest species of different orders. Pest Manag. Sci..

[60] Fitches E., Wiles D., Douglas A.E., Hinchliffe G., Audsley N., Gatehouse J.A. (2008). The insecticidal activity of recombinant garlic lectins towards aphids. Insect Biochem. Mol. Biol..

[61] Godfray H.C.J., Beddington J.R., Crute I.R., Haddad L., Lawrence D., Muir J.F., Pretty J., Robinson S., Thomas S.M., Toulmin C. (2010). Food security: the challenge of feeding 9 billion people. Science.

[62] Kostyukovsky M., Shaaya E., Ishaaya I., Palli S.R., Horowitz A.R. (2013). Advanced Methods for Controlling. Insect Pests in Dry Food. Advanced Technologies for Managing Insect Pests SE-14.

[63] Self M., Turner J.A. (2009). Market access for New Zealand forest products: An economic and environmental case for development of alternative phytosanitary treatments. New Zeal. J. For. Sci..

[64] Horne P.A., Page J., Nicholson C. (2008). When will integrated pest management strategies be adopted? Example of the development and implementation of integrated pest management strategies in cropping systems in Victoria. Aust. J. Exp. Agric..

[65] Gentz M.C. (2009). A review of chemical control options for invasive social insects in island ecosystems. J. Appl. Entomol..

[66] Brockerhoff E., Liebhold A., Richardson B., Suckling D. (2010). Eradication of invasive forest insects: Concepts, methods, costs and benefits. New Zeal. J. For. Sci..

[67] Pimentel D., Zuniga R., Morrison D. (2005). Update on the environmental and economic costs associated with alien-invasive species in the United States. Ecol. Econ..

[68] Mooney H.A., Cleland E.E. (2001). The evolutionary impact of invasive species. Proc. Natl. Acad. Sci. USA.

[69] Peck S.L. (2001). Antibiotic and insecticide resistance modeling—Is it time to start talking?. Trends Microbiol..

