# Supplementary material for: Resistance is not Futile: It Shapes Insecticide Discovery
Source: Insects. 2014 Jan 23;5(1):227–42. doi: 10.3390/insects5010227 (PMC4592624; doi:10.3390/insects5010227)
Supplement: Supplementary File 1 [file insects-05-00227-s001.pdf]

Table S1. Classification of insecticides from IRAC.

Group		Subgroup	Chemical Class/Example AI
1	Acetylcholinesterase inhibitor	A	Carbamates
		B	Organophosphates
2	GABA-gated chloride channel antagonists	A	Cyclodiene organochlorines
		B	Phenylpyrazoles (fiproles)
3	Sodium channel modulators	A	Pyrethroids, pyrethrins
		B	DDT, methoxychlor
4	Nicotinic acetylcholine receptor agonists	A	Neonicotinoids
		B	Nicotine
		C	Sulfoxaflor
5	Nicotinic acetylcholine receptor allosteric activators		Spinosyns
6	Chloride channel activators		Avermectins, milbemycins
7	Juvenile hormone mimics	A	Juvenile hormone analogs
		B	Fenoxycarb
		C	Pyriproxyfen
8	Miscellaneous nonspecific (multi-site) inhibitors	A	Alkyl halides
		B	Chloropicrin
		C	Sulfuryl fluoride
		D	Borates
		E	Tartar emetic
9	Selective homopteran feeding blockers	A	Pymetrozine
		B	Flonicamid
10	Mite growth inhibitors	A	Clofentezine
		B	Etoxazole
11	Microbial disruptor of insect midgut membranes	A	*Bacillus thuringiensis* (incl. insecticidal proteins)
		B	*Bacillus spaericus* (incl. insecticidal proteins)
12	Inhibitors of mitochondrial ATP synthase	A	Diafenthiuron
		B	Organotin miticides
		C	Propargite
		D	Tetradifon
13	Uncouplers of oxidative phosphorylation via disruption of proton gradient	A	Chlorfenapyr
		B	Dinitro-*ortho*-cresol (DNOC)
		C	Sulfuramid
14	Nicotinic acetylcholine receptor channel blockers		Nereistoxin analogs
15	Inhibitors of chitin biosynthesis, type 0		Benzoylureas
16	Inhibitors of chitin biosynthesis, type 1		Buprofezin
17	Moulting disruptor, Diptera		Cyromazine
18	Ecdysone receptor agonists		Diacylhydrazines
19	Octopaminergic receptor agonists		Amitraz
20	Mitochondrial Complex III electron transport inhibitors	A	Hydramethylnon
		B	Acequinocyl
		C	Fluacrypyrim
21	Mitochondrial Complex I electron transport inhibitors	A	Mitochondrial electron transport inhibitor (METI) acaricides and insecticides
		B	Rotenone
22	Voltage-dependent sodium channel blockers	A	Indoxacarb
		B	Metaflumizone
23	Inhibitors of acetyl CoA carboxylase - Lipid synthesis, growth regulation		Tetronic and tetramic acid derivatives
24	Mitochondrial Complex IV electron transport inhibitors	A	Aluminum/Calcium/Zinc phosphide, phosphine
		B	Cyanide
25	Mitochondrial Complex II electron transport inhibitors		Pyrazole
28	Ryanodine receptor modulators		Diamides
UN	Compounds of unknown or uncertain mode of action	A	Azadirachtin
		B	Bifenazate
		C	Promopropylate
		D	Chinomethionat
		E	Cryolite
		F	Dicofol
		G	Pyridalyl
		H	Pyrifluquinazon
