# Supplementary material for: Resistance is not Futile: It Shapes Insecticide Discovery
Source: Insects. 2014 Jan 23;5(1):227–42. doi: 10.3390/insects5010227 (PMC4592624; doi:10.3390/insects5010227)
Supplement: Supplementary File 2 [file insects-05-00227-s002.pdf]

Table S2. The number of invasive insect species in North America.

Order	Family	Species
Blattodea	Blattellidae	2
	Blattidae	1
Coleoptera	Anobiidae	1
	Bostrichidae	1
	Brachyceridae	1
	Brentidae	1
	Buprestidae	4
	Cerambycidae	35
	Chrysomelidae	15
	Coccinellidae	4
	Cryptophagidae	1
	Curculionidae	99
	Dermestidae	1
	Elateridae	5
	Nitidulidae	1
	Scarabaeidae	13
	Tenebrionidae	1
Collembola	Sminthuridae	1
Diptera	Agromyzidae	3
	Cecidomyiidae	4
	Culicidae	2
	Drosophilidae	1
	Muscidae	1
	Oestridae	1
	Sarcophagidae	1
	Tephritidae	22
	Tipulidae	3
Hemiptera	Adelgidae	3
	Aleyrodidae	8
	Alydidae	1
	Aphididae	6
	Aradidae	1
	Blissidae	1
	Cicadellidae	6
	Coccidae	10
	Coreidae	1
	Delphacidae	1
	Diaspididae	22
	Eriococcidae	3
	Kerriidae	1
	Lygaeidae	3
	Margarodidae	1
	Miridae	2
	Oxycarenidae	1
	Pentatomidae	4
	Phylloxeridae	1
	Piesmatidae	1
	Plataspidae	1
	Pseudococcidae	9
	Psyllidae	2
	Pyrrhocoridae	1
	Scutelleridae	1

Order	Family	Species
Hymenoptera	Agaonidae	2
	Aphelinidae	1
	Apidae	3
	Chrysididae	1
	Cynipidae	1
	Diprionidae	2
	Formicidae	9
	Megachilidae	1
	Pamphiliidae	1
	Siricidae	6
	Tenthredinidae	2
	Vespidae	4
Isoptera	Kalotermitidae	3
	Rhinotermitidae	1
	Termitidae	1
	Termopsidae	2
Lepidoptera	Agonoxenidae	1
	Carposinidae	1
	Choreutidae	1
	Cossidae	2
	Crambidae	9
	Elachistidae	1
	Erebidae	2
	Galacticidae	1
	Gelechiidae	6
	Geometridae	3
	Gracillariidae	4
	Lasiocampidae	5
	Limacodidae	1
	Lycaenidae	1
	Lymantriidae	9
	Lyonetiidae	1
	Noctuidae	14
	Nolidae	1
	Notodontidae	2
	Opostegidae	1
	Papilionidae	1
	Pieridae	2
	Plutellidae	1
	Psychidae	1
	Pyralidae	2
	Saturniidae	2
	Sesiidae	2
	Sphingidae	1
	Tineidae	1
	Tortricidae	26
	Yponomeutidae	5
Odonata	Libellulidae	4
Orthoptera	Gryllotalpidae	2
	Tettigoniidae	1
Thysanoptera	Phlaeothripidae	1
	Thripidae	9
	**Total**	483
